# Does spatiotemporal nutrient variation allow more species to coexist?

**DOI:** 10.1007/s00442-020-04768-9

**Published:** 2020-10-24

**Authors:** Josie Antonucci Di Carvalho, Stephen A. Wickham

**Affiliations:** Department of Ecology and Evolution, University of Salzburg, Hellbrunnerstrasse 34, 5020 Salzburg, Austria

**Keywords:** Metacommunity, Phytoplankton, Zooplankton, Connectivity, Eutrophication

## Abstract

Temporal heterogeneity in nutrient availability is known to increase phytoplankton diversity by allowing more species to coexist under different resource niches. Spatial heterogeneity has also been positively correlated with species diversity. Here we investigated how temporal and spatial differences in nutrient addition together impact biodiversity in metacommunities varying in the degree of connectivity among the patches. We used a microcosm experimental design to test two spatiotemporal ways of supplying nutrients: synchronously (nutrients were added regionally—to all four patches at the same time) and asynchronously (nutrients were added locally—to a different patch each time), combined with two different degrees of connectivity among the patches (low or high connectivity). We used three species of algae and one species of cyanobacteria as the primary producers; and five ciliate and two rotifer species as the grazers. We expected higher diversity in metacommunities receiving an asynchronous nutrient supply, assuming stronger development of heterogeneous patches with this condition rather than with synchronous nutrient supply. This result was expected, however, to be dependent on the degree of connectivity among patches. We found significant effects of nutrient addition in both groups of organisms. Phytoplankton diversity increased until the fourth week (transiently) and zooplankton richness was persistently higher under asynchronous nutrient addition. Our results were consistent with our hypothesis that asynchronicity in nutrient supply would create a more favorable condition for species to co-occur. However, this effect was, in part, transient and was not influenced by the degree of connectivity.

**Electronic supplementary material:**

The online version of this article (10.1007/s00442-020-04768-9) contains supplementary material, which is available to authorized users.

## Introduction

As habitat fragmentation and biodiversity loss continues unabated, the role of metacommunities in preserving diversity and the accompanying ecosystem services is becoming ever more relevant. In unconnected fragments, extinctions can occur remarkably quickly, but if patches are interconnected to an appropriate degree, forming a metacommunity, local extinction can be countered by species dispersal, resulting in regional survival (Holyoak and Lawler 1996; Holyoak 2000; Gibson et al. 2013). Theory has proposed, and experiments shown, that high degrees of connectivity allows patches to become synchronized and local extinctions occur in all patches simultaneously, while at lower levels of connectivity, local extinctions can be countered by immigration from the regional species pool (Taylor 1990; Limberger and Wickham 2012a). What constitutes “low” or “high” degree of connectivity is clearly dependent on the context of the community and habitat. In a metacommunity where species dispersal is low, strong competitors or predators allow for longer coexistence in the different interconnected localities (Leibold and Chase 2018). Conversely, high dispersal rates can either increase species coexistence by allowing species persistence even in unfavorable localities (Loreau and Mouquet 1999), or decrease diversity when superior competitors or generalist predators are widespread over all patches (Loreau and Mouquet 1999; Holyoak et al. 2005; Limberger and Wickham 2011). In an experimental study that directly manipulated connectivity, both regional diversity and local dissimilarity were highest in metacommunities with low connectivity, as different species could dominate in different patches (Limberger and Wickham 2012b).

Of the many anthropogenic stressors impacting biodiversity, eutrophication is one of the most widespread and relevant, particularly for aquatic communities (Smith and Schindler 2009). Anthropogenic eutrophication is a long-term problem at a world-wide scale, with either point discharge stemming from insufficient water treatment from towns and cities, or broader nutrient runoff, primarily stemming from agricultural landscapes (Glibert 2017; Schneider et al. 2018). Increased nutrient loading can lead to reduced biodiversity through such mechanisms as increased shading, reduced niche dimensions, or through mortality due to hypoxia or toxic algae (Anderson et al. 2002; Diaz and Rosenberg 2008; Hautier et al. 2009; Smith and Schindler 2009; Harpole et al. 2011). However, creating temporal variability by pulsing nutrients weekly has been shown to increase algal diversity compared to a continuous addition (Sommer 1985). This effect can also be seen in a metacommunity context, where temporal nutrient heterogeneity has a positive influence on species diversity, in both primary producers and their grazers, most likely due to temporal nutrient heterogeneity allowing species with varying survival abilities to coexist (Roelke and Spatharis 2015; Smeti et al. 2016; Di Carvalho and Wickham 2019; Papanikolopoulou et al. 2018). Fluctuations of nutrient availability can potentially also impact ecosystem functions (Roelke and Spatharis 2015; Smeti et al. 2016) and these impacts can have positive or negative effects on biodiversity, depending on the original state of the ecosystem productivity (Heino 2013).

Within a metacommunity context, whether eutrophication has negative or positive effects on biodiversity may be dependent upon not only the temporal scale at which it occurs, but also the spatial scale. If eutrophication occurs at a regional scale, impacting all patches equally, then the negative impacts seen in field studies may dominate, reducing diversity. Conversely, if eutrophication is restricted to only a few patches within the metacommunity, spatial heterogeneity may be generated at the regional scale, allowing niche partitioning and species sorting to occur. The spatial heterogeneity of nutrient availability among sites will result in lower local and higher regional diversity. This scenario has been invoked to explain high beta diversity in a region with lakes impacted to varying degrees by eutrophication (Davies et al. 2009; Maloufi et al. 2016).

While many studies have investigated spatial heterogeneity in a metacommunity context (Holt 1985; Reynolds et al. 2007; Matthiessen et al. 2010; Pedruski and Arnott 2011; Limberger et al. 2017), few studies examined the interactive effects of spatiotemporal heterogeneity and connectivity (but see Carrara et al. (2012)) with even less research considering spatiotemporal variation of nutrients. Our experimental work aimed to reduce this shortage by elucidating how temporal and spatial nutrient variability (spatiotemporal heterogeneity) affect biodiversity in a metacommunity landscape varying in its connectivity level. To investigate the link between species diversity and ecosystem functioning, we also measured the resource use efficiency (RUE) in our microcosms. The quantification of new biomass realized from supplied resources can explain individual species contribution to the biomass production (Hodapp et al. 2019). This concept acknowledges the importance of considering species traits beyond species diversity in analyzing ecology processes.

Our metacommunities were composed of four microcosms (patches) interconnected by tubes which allowed for either low or high connectivity depending on the opening times. In a pulsed fashion, nutrients were added either synchronously—at the regional scale, resulting in spatially and temporally similar patches; or asynchronously—at the local scale, with nutrients varying spatially and temporally among the patches.

With four different treatment combinations—high connectivity and synchronous nutrient addition (HS); high connectivity and asynchronous nutrient addition (HA); low connectivity and synchronous nutrient addition (LS); low connectivity and asynchronous nutrient addition (LA)—we investigated the following hypotheses:

### h1

Diversity will be higher in metacommunities receiving asynchronous pulses of nutrients, as spatiotemporal nutrient heterogeneity will allow more species to coexist at the regional scale;

### h2

The effects of nutrient addition should be dependent, however, on the degree of connectivity between patches. At high connectivity, diversity will not be different between metacommunities with synchronous or asynchronous pulses, since the high species dispersal would overcome the heterogeneity among patches, homogenizing the entire metacommunity, allowing for the best competitors to dominate;

### h3

We further assumed that higher beta diversity would be promoted by asynchronous nutrient addition, and the dissimilarities among the patches would be even greater in the low connectivity level (LA);

### h4

We finally predicted that the higher diversity in the LA treatment would also result in higher resource use efficiency (RUE), as measured by the ratio of predator to prey biomass.

## Materials and methods

### Species

The experimental community was composed of two trophic levels: primary producers and grazers. The former were represented by three algae and one cyanobacteria species: *Desmodesmus abundans, Cryptomonas* sp. Strain SAG 26.80*, Chlamydomonas* sp. and *Synechococcus* sp. The grazer community was composed of five ciliate species and two rotifers species: *Coleps hirtus hirtis, Paramecium bursaria, Halteria* sp.*, Stylonychia* sp.*, Cyclidium* sp.*, Lepadella* sp. and *Synchaeta oblonga*. The four prey species were chosen based on differences in nutritional quality for grazers. Cyanobacteria are phosphorous-rich but a poor food resource for grazers because of their deficiency in essential omega-3 fatty acids (Martin-Creuzburg and von Elert 2009). In comparison, *Desmodesmus abundans* and *Chlamydomonas* sp. are better food quality, except for their relatively low concentrations of highly unsaturated fatty acids (Taipale et al. 2016; Peltomaa et al. 2017). Cryptomonads, however, are rich in lipid composition, promoting better grazer growth (Skogstad et al. 1987; Vanormelingen et al. 2009; Taipale et al. 2018). Ciliates, including species used in this study, have markedly higher growth rates when grown monoxenically on *Cryptomonas* compared to *Synechococcus* (Wickham and Wimmer 2019).

Microzooplankton species were isolated from ponds in the city of Salzburg, Austria, and are species that coexist with one another in natural systems. Freshwater samples were collected using a plankton net with a 30 µm mesh size, during summer 2015. Species were isolated and transferred to 6-well plates with medium (Volvic© water + algae) for culturing. The autotrophic species had been originally obtained from the culture collection at Göttingen (SAG) and had been in culture in our lab for several years.

Each bottle started with the same species of autotrophs and heterotrophs in equivalent biomass. Grazer and primary producer biomass were estimated using carbon conversion factors from the literature (Rocha and Duncan 1985; Stemberger and Gilbert 1987; Putt and Stoecker 1989).

### Experimental design

A metacommunity was represented by four 125 ml polycarbonate bottles interconnected with silicon tubes (0.5 cm inner diameter and 20 cm length), allowing active dispersal of species. All ciliates, rotifers and two algae species, excluding the non-motile species *Desmodesmus abundans* and *Synechococcus* sp.*,* were able to actively disperse among the bottles. Each bottle was connected with its other two neighboring bottles, in the form of a square. Two different factors were manipulated in this experiment (Online Resource Fig. S1):

### Connectivity

The difference between high and low connectivity was manipulated with the opening time of the tubes. Each tube was clamped to prevent dispersal. The clamps were opened continuously for 48 h/week in the metacommunities with high connectivity and continuously for 4 h/week in the metacommunities with low connectivity. The design was chosen based on preliminary experiments, in which the dispersal abilities of the zooplankton species were measured. In these experiments, two bottles were connected by a silicon tube of 20 cm length. The species’ dispersal rates were measured as the time needed for each species to reach an empty patch. One patch was seeded with ca. 500 individuals of each species and the connected patch contained only medium. This patch was sampled every four hours until all species appeared. *Coleps hirtus hirtis* and *Paramecium bursaria* migrated to the second patch within 4 h, while the other species were found in the second patch 20 h after the beginning of the experiment.

### Nutrient addition

We added phosphorus and nitrogen in the Redfield (1958) 16:1 ratio at the, respectively, rates: *p*: 0,016 µmol P L^−1^ day^−1^; *N*: 0,258 µmol N L^−1^ day^−1^. Nutrients were supplied once every 5 days (a pulsed nutrient addition) either synchronously or asynchronously. The synchronous enrichment was characterized by the simultaneous addition of nutrients to all four patches, every 5 days, whereas the asynchronous supply was defined by the addition of the same total amount of nutrients but to only one patch, switching the patches receiving the nutrients each 5 days (one patch after the other, consecutively). In this way, spatiotemporal variability in nutrient addition was created, with each patch receiving two nutrients pulses by the end of the experiment. To add the same total amount of nutrients, 8 ml week^−1^ of nutrients were added to each patch of a metacommunity under synchronous nutrient addition; or 32 ml week^−1^ of nutrients were added to one patch, every 5 days, consecutively in the asynchronous treatment. Therefore, every week, one patch of the asynchronous treatment was receiving much larger amount of nutrients than the patches in the synchronous treatment.

We tested four treatment combinations: high connectivity and synchronous nutrient addition —HS; high connectivity and asynchronous nutrient addition—HA; low connectivity and synchronous nutrient addition—LS; low connectivity and asynchronous nutrient addition—LA. Each treatment was replicated three times, resulting in 12 sets of 4-bottle metacommunities, totaling 48 bottles for the whole experiment. The initial volume of each metacommunity patch was 80 ml, with 10 ml additional volume in each tube. We first filled the bottles with Volvic© mineral water, then we added nutrients, and finally, the organisms. The experiment was conducted in a laboratory with constant temperature of 20 °C on a 12:12 light:dark cycle with light intensity high enough to ensure algal growth (~ 200 µE).

The experiment was carried out for seven weeks and was sampled weekly, with 10 ml taken from each metacommunity bottle. The samples were fixed with Lugol’s iodine solution. The volume of sample analyzed varied between 1 and 3 ml, depending on the abundance of species observed. The individuals were counted under an inverted microscope.

### Statistical analyses

All statistical analyses were performed with R (R version 3.4.1; see Online Resource Table S1 for more details).

Biodiversity of zooplankton and phytoplankton were measured separately as a Shannon Wiener index at local and regional scales. To estimate the local diversity, the Shannon index was calculated for each patch of a metacommunity and then averaged over the four patches. Regional diversity was based on the mean abundance of an entire metacommunity to calculate the Shannon index. Likewise, richness and evenness were calculated for both groups of organisms at both local and regional scales. Evenness was calculated as a Pielou’s index. Beta diversity was calculated as Bray Curtis dissimilarities using the abundance data in each local community.

The absolute biomass of zooplankton and phytoplankton were calculated locally, for each patch, and then averaged among the four patches of a metacommunity. Resource use efficiency (RUE) was calculated as the biomass ratio between grazers and primary producers (grazer biomass/primary producer biomass). The biomass ratio was estimated for each of the four treatment combinations, over the 7 weeks of experiment. We further investigated possible significant correlations between RUE and diversity measures (Shannon index, richness and evenness) and time.

The effects of nutrient addition and connectivity on Shannon diversity, richness, evenness, Bray Curtis dissimilarity and biomass were tested using two-way ANOVA with repeated measures, using time as a within subject variable and nutrient addition and connectivity as the two between subject variables. The *p* values were evaluated considering the significance threshold at 0.05. When interactions between treatment factors were significant, Tukey´s post-hoc tests were applied to better investigate the relations encountered.

## Results

### Diversity

**Fig. 1:**
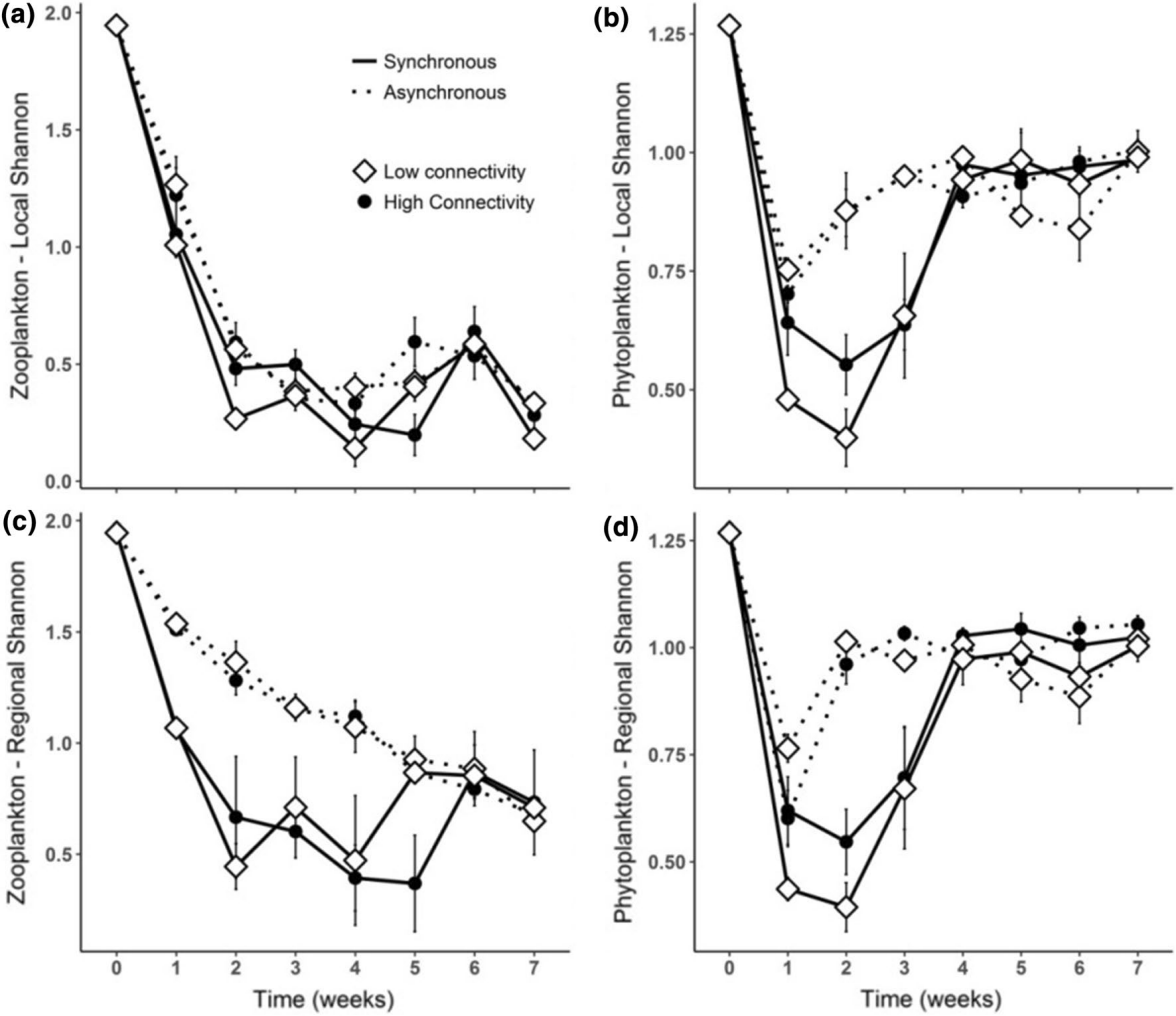
Shannon diversity of phytoplankton and zooplankton. **a**, **b** Represents the local scale responses; **c**, **d** represents the regional scale responses. In the graphs, low connectivity is represented by opened diamonds; high connectivity is represented by closed circles; synchronous nutrient addition is represented by solid lines and asynchronous nutrient addition is represented by dotted lines. Values are mean ± SE, *n* = 3

Primary producers’ and grazers’ diversity strongly responded to nutrient addition at both scales (Fig. 1), with higher significant responses at the regional scale (Tables 1, 2). While these effects were clearly observed, the different degrees of connectivity did not influence any of the organism groups during the experiment. Furthermore, time strongly shaped the nutrient addition effects in all cases (Tables 1, 2).

**Table 1:** Two way ANOVA with repeated measures

	Nutrient		Connectivity		Nutrient:connectivity		Time		Nutrient:time		Connectivity:time	
	*F*_1,8_	*p*	*F*_1,8_	*p*	*F*_1,8_	*p*	*F*_7,56_	*p*	*F*_7,56_	*p*	*F*_7,56_	*p*
Shannon (*α*)	16.0	**0.003**	1.09	0.326	0.43	0.532	8.44	** < 0.001**	16.25	** < 0.001**	0.96	0.471
Shannon (*γ*)	2.18	**0.002**	2.41	0.159	1.57	0.245	72.02	** < 0.001**	1.82	** < 0.001**	0.59	0.756
Richness (*α*)	0.85	0.383	0.49	0.501	1.08	0.33	8.44	** < 0.001**	2.12	0.057	1.21	0.312
Richness (*γ*)	0.85	0.383	0.49	0.501	1.08	0.33	8.44	** < 0.001**	2.12	0.057	1.21	0.312
Evenness (*α*)	16.0	**0.003**	1.09	0.326	0.43	0.532	43.68	** < 0.001**	16.25	** < 0.001**	0.96	0.471
Evenness (*γ*)	2.18	**0.002**	2.41	0.159	1.57	0.245	42.04	** < 0.001**	1.82	** < 0.001**	0.59	0.756
Biomass	2.61	** < 0.001**	0.44	0.5261	2.06	0.189	98.15	** < 0.001**	4.11	0.077	0.25	0.629
Bray Curtis	10.1	**0.002**	0.66	0.42	0.05	0.824	25.13	** < 0.001**	5.92	** < 0.001**	1.14	0.348

Testing the effects of nutrient addition, connectivity and time on Shannon diversity, richness and biomass of phytoplankton community, at regional and local scales. Significant *p* values are in bold

*Shannon = Shannon Wiener diversity; *γ* = regional; *α* = local

**Table 2:** Two way ANOVA with repeated measures. Testing the effects of nutrient addition, connectivity and time on Shannon diversity, richness and biomass of zooplankton community, at regional and local scales

	Nutrient		Connectivity		Nutrient:connectivity		Time		Nutrient:time		Connectivity:time	
	*F*_1,8_	*p*	*F*_1,8_	*p*	*F*_1,8_	*p*	*F*_7,56_	*p*	*F*_7,56_	*p*	*F*_7,56_	*p*
Shannon (*α*)	7.67	**0.024**	0.681	0.433	0.54	0.482	366.7	** < 0.001**	4.02	**0.001**	0.55	0.789
Shannon (*γ*)	33.07	** < 0.001**	0.47	0.512	0.08	0.788	54.27	** < 0.001**	8.17	** < 0.001**	0.85	0.55
Richness (*α*)	2.31	0.167	0.03	0.870	0.26	0.626	409.01	** < 0.001**	10.5	** < 0.001**	0.50	0.828
Richness (*γ*)	20.58	**0.002**	0.06	0.811	0.33	0.579	7.45	** < 0.001**	12.8	** < 0.001**	0.32	0.941
Evenness (*α*)	0.24	0.632	0.07	0.799	0.35	0.567	23.6	** < 0.001**	2.12	**0.06**	0.59	0.756
Evenness (*γ*)	6.59	**0.033**	0.72	0.421	1.09	0.326	8.56	** < 0.001**	2.87	**0.01**	0.43	0.878
Biomass	0.95	0.358	1.47	0.260	0.84	0.386	9312.4	** < 0.001**	5.19	**0.05**	0.2	0.178
Bray curtis	29.98	** < 0.001**	0.19	0.659	0.61	0.437	37.4	** < 0.001**	3.92	**0.001**	0.47	0.854

Significant *p* values are in bold

*Shannon = Shannon Wiener diversity; *γ* = regional; *α* = local

Similar patterns of phytoplankton diversity were observed at local and regional scales (Fig. 1b, d). Despite the strong decrease in diversity during the initial phase of the experiment, phytoplankton diversity showed a faster recovery under asynchronous nutrient addition (LA and HA) than under the synchronous supply (LS and HS). However, these effects were transitory, with differences in the nutrient supply converging on the fourth week.

In contrast to the phytoplankton diversity responses, zooplankton diversity strongly differed between local and regional scales (Fig. 1a, c). Locally, grazer diversity decreased sharply until the second week of the experiment, not showing any important recovery afterwards (Fig. 1a). Conversely, at the regional scale higher diversity was maintained under asynchronous nutrient addition for a longer period, compared to the lower zooplankton diversity encountered in LS and HS treatments (Fig. 1c; Table 2; *F*_1,8_ = 33.07, *p* < 0.001). Similar to the phytoplankton responses, zooplankton diversity under asynchronous and synchronous nutrient supply converged on the fifth week (Fig. 1c).

While grazer richness was affected by nutrient addition, mainly at the regional scale (Table 2; *F*_1,8_ = 20.58, *p* = 0.002), primary producers did not show any change in richness due to the treatments (Table 1). Phytoplankton initial richness was composed of four species, declining to three species after the first week of the experiment, when *Cryptomonas* sp. went extinct at both the local and regional scale. However, autotrophs’ evenness was positively affected by nutrient addition (Table 1), with equal Pielou’s and Shannon indexes, indicating that diversity of primary producers was only due to evenness. For these reasons, the results of phytoplankton richness and evenness are not depicted.

Grazer richness and evenness were influenced by nutrient addition mainly at the regional scale, but also at the local scale, with a time dependency (Table 2). Locally, zooplankton richness was maintained until the second week, dropping constantly afterwards (Online Resource Fig. S2a). In contrast, regional richness remained higher under asynchronous nutrient addition than under synchronous nutrition (Online Resource Fig. S2c). The positive effect of asynchronous nutrient addition was also observed for the grazers’ evenness at the regional scale; however, the dissimilarities between the nutrient treatments were transitory, converging on the fifth week (Online Resource Fig. S2d).

**Fig. 2:**
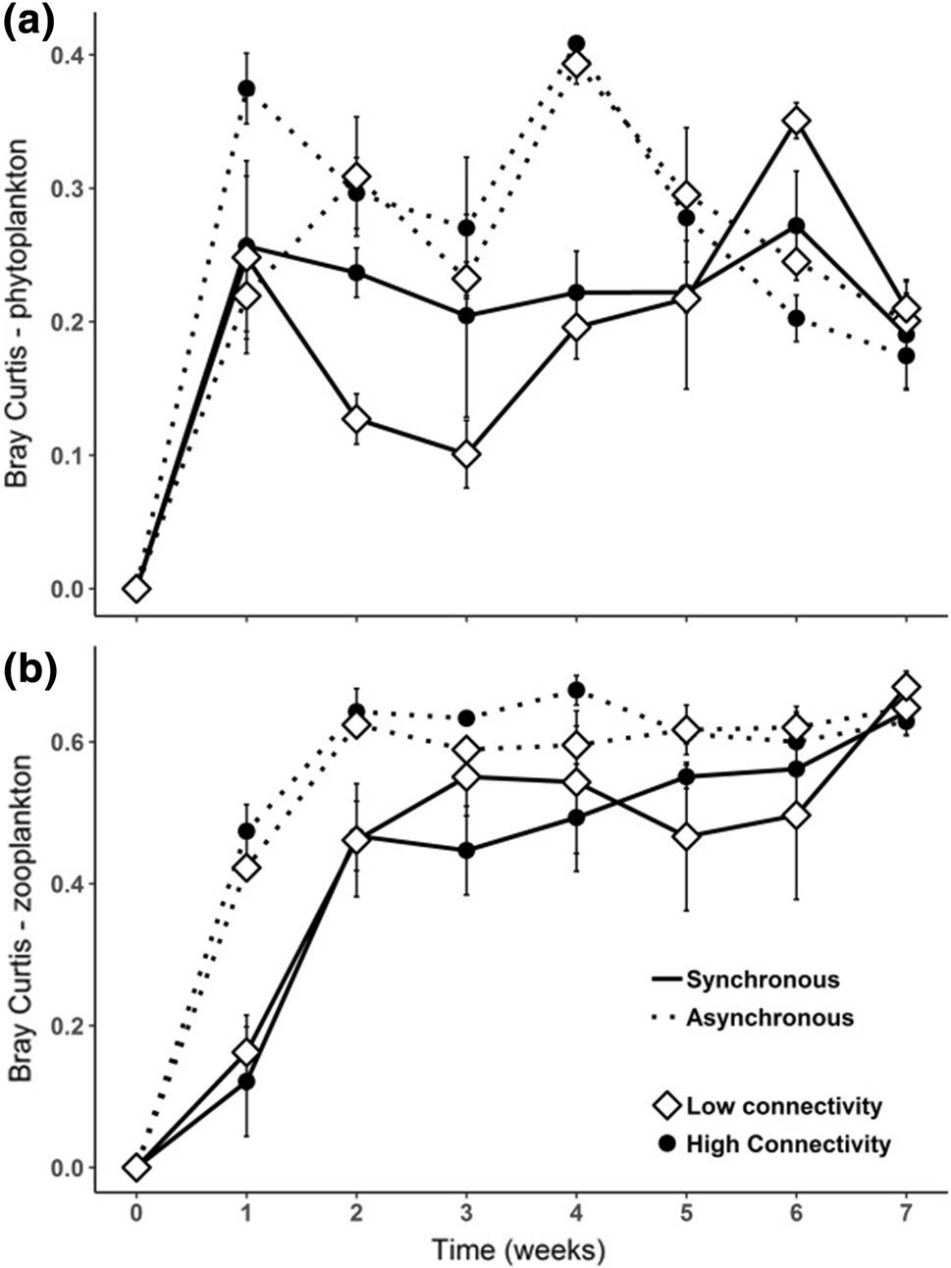
Phytoplankton (**a**) and zooplankton (**b**) beta diversity measured as Bray Curtis dissimilarities among the local patches. Low connectivity is represented by opened diamonds; high connectivity is represented by closed circles; synchronous nutrient addition is represented by solid lines and asynchronous nutrient addition is represented by dotted lines. Values are mean ± SE, *n* = 3

Phytoplankton and zooplankton beta diversities were significantly affected by the different nutrient additions (Tables 1, 2), showing similar patterns to Shannon diversity, richness and evenness. Asynchronous nutrient addition promoted greater dissimilarity among patches most of the time, without showing significant effects of high or low connectivity (Fig. 2a, b). Moreover, Bray Curtis dissimilarities fluctuated more over time in primary producers than in grazers, which showed a smoother trend.

### Species abundance, biomass and the resource use efficiency

**Fig. 3:**
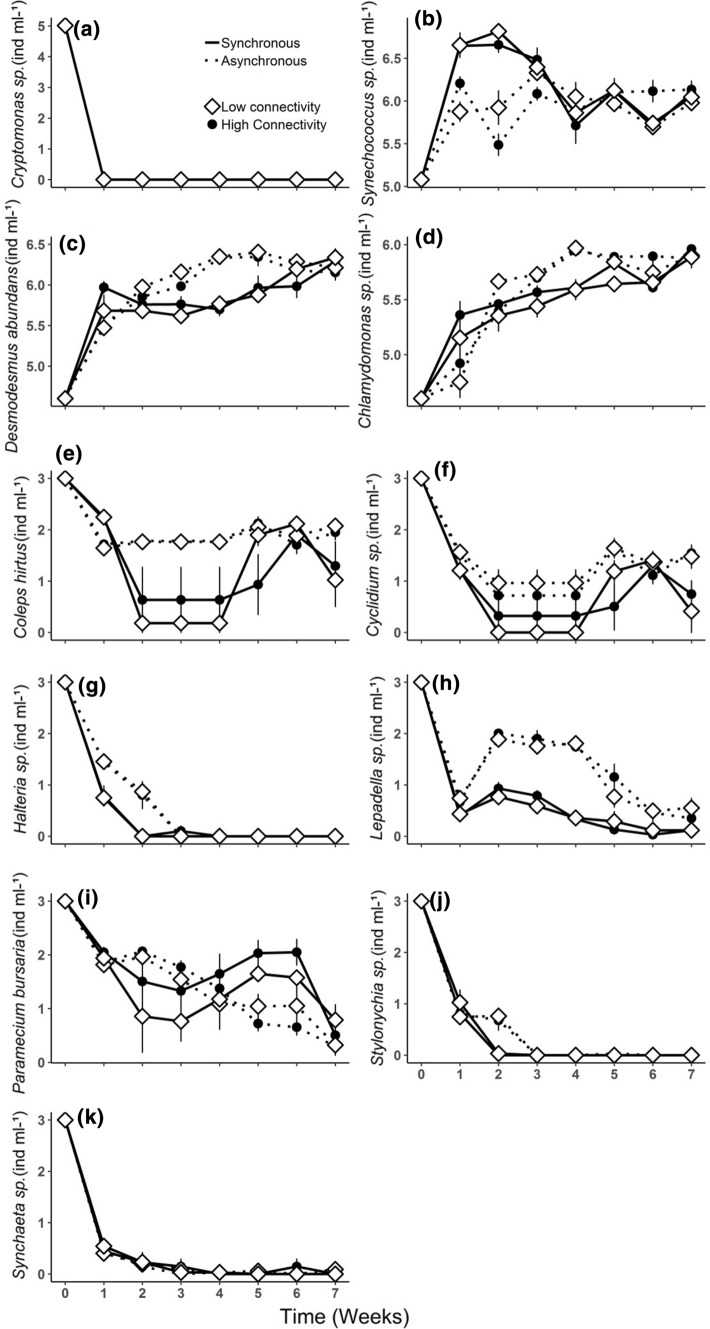
Abundance of each phytoplankton and zooplankton species over the experiment time. **a** *Cryptomonas* sp., **b** *Synechococcus* sp., **c** *Desmodesmus abundans*, **d** *Chlamydomonas* sp., **e** *Coleps hirtus*, **f** *Cyclidium* sp., **g** *Halteria* sp., **h** *Lepadella* sp., **i** *Paramecium bursaria*, **j** *Stylonychia* sp., **k** *Synchaeta* sp.. In the graphs, low connectivity is represented by opened diamonds; high connectivity is represented by closed circles; synchronous nutrient addition is represented by solid lines and asynchronous nutrient addition is represented by dotted lines. Values are mean ± SE, *n* = 3. Note log scale used in panel (log_10_ + 1)

Similar to the results on diversity, the biomass of phytoplankton and zooplankton were strongly affected by the mode of nutrient supply and by time, without significant effects of connectivity (Tables 1, 2). The highest absolute biomass over time was encountered in metacommunities with asynchronous nutrient addition (Fig. Online Resource Fig. S3) and the main contributing species was the green algae *Chlamydomonas* sp. This species, together with the green algae *Desmodesmus abundans*, were more abundant in HA and LA than in HS and LS treatments (Fig. 3c, d). Since *Cryptomonas* sp. disappeared on the first week of the experiment (Fig. 3a), this species did not contribute to the total biomass of primary producers. The LA and HA treatments promoted the highest abundance over time of *Desmodesmus abundans* and *Chlamydomonas* sp. abundances (Fig. 3c, d), whereas LS and HS promoted highest abundance over time of *Synechoccocus* sp.. In the initial phase of the experiment, the cyanobacteria had a greater increase in the synchronous nutrient addition treatment than in the asynchronous addition, reaching their maximum abundance on the second week (Fig. 3b). This fast increase then ceased, and the dissimilar effects between synchronous and asynchronous supply converged on the fourth week (Fig. 3b).

As with phytoplankton, the highest absolute zooplankton biomass was encountered in metacommunities with asynchronous nutrient addition (Online Resource Fig. S4). The main contributing species for the total biomass was the *Paramecium bursaria*, in all four treatment combinations. Interestingly, this species showed an important switch in abundance during the middle period of the experiment. It decreased in the metacommunities with asynchronous enrichment but increased in the metacommunities with synchronous enrichment (Fig. 3i). *Halteria* sp. and *Stylonychia* sp. decreased in abundance from the beginning of the experiment onwards, in all four treatments, disappearing after the third week. However, this decrease was faster in HS and LS than in HA and LA treatments (Fig. 3g, j). In general, all the grazers species, except for *Synchaeta oblonga*, which was unaffected by the treatments, showed higher abundance over time under asynchronous nutrient supply than under synchronous enrichment.

**Fig. 4:**
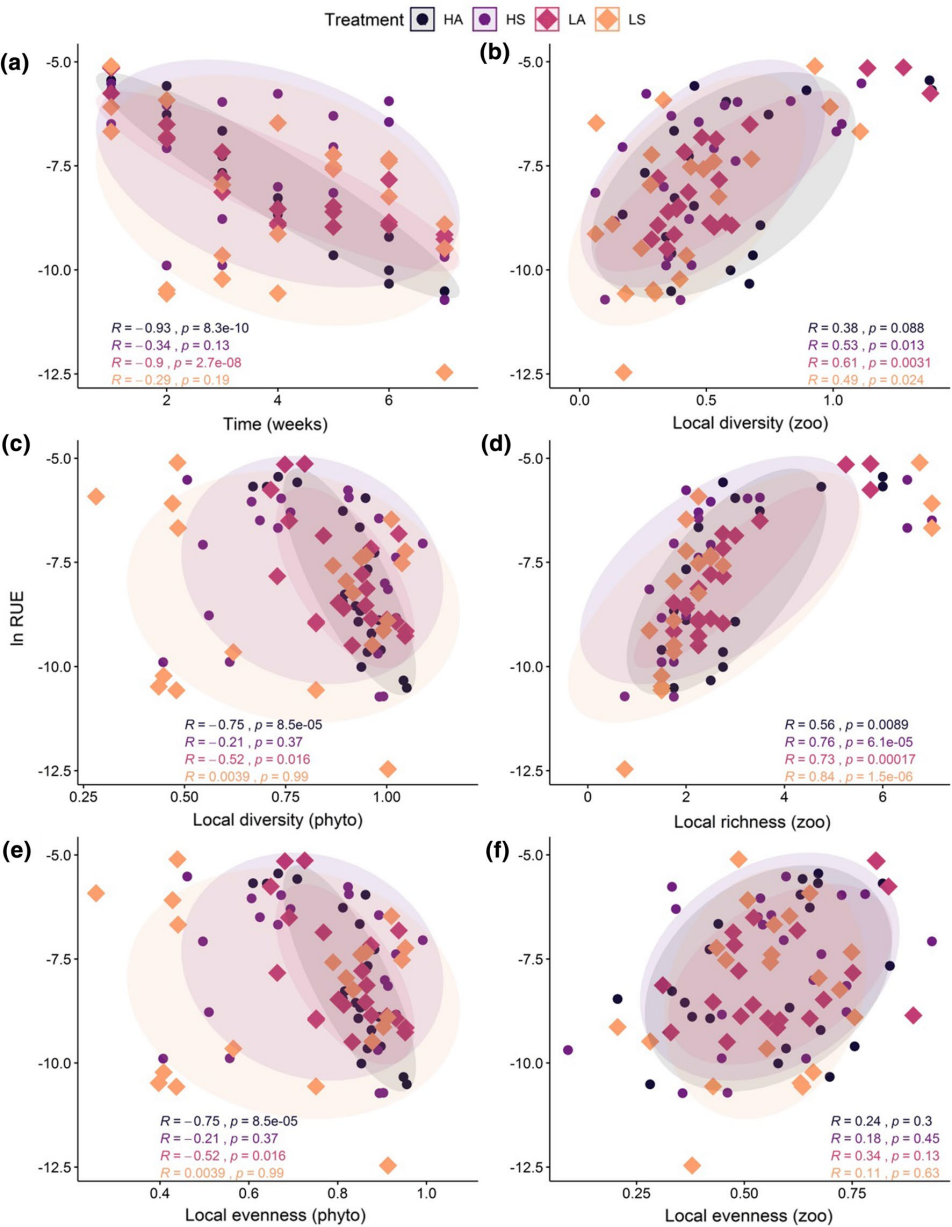
Correlation between **a** resource use efficiency and time; **b** resource use efficiency and zooplankton local Shannon diversity; **c** resource use efficiency and phytoplankton local Shannon diversity; **d** resource use efficiency and local zooplankton richness; **e** resource use efficiency and local phytoplankton evenness; **f** resource use efficiency and zooplankton local evenness. RUE was measured as zooplankton biomass per phytoplankton biomass. Spearman correlation values (*r*) are represented for each treatment combination. Values are mean ± SE, *n* = 3. A color version of the figure is available online. The circles refer to 95% confidence ellipse. *p < 0.05, **p < 0.01, ***p < 0.001

Increasing zooplankton diversity and richness resulted in higher zooplankton biomass, relative to phytoplankton biomass. The linear relationships between resource use efficiency (RUE) and zooplankton diversity (Fig. 4b, d) showed a significant positive correlation, in nearly all four treatment combinations, excepted for the HA treatment which did not show significant effects (Fig. 4b). The correlation between RUE and zooplankton richness was greater than the correlation with Shannon diversity, with positive relationships in all four treatment combinations (Fig. 4d), whereas the correlation with zooplankton evenness was not significant in any treatment (Fig. 4f). Conversely, the RUE was inversely correlated with the phytoplankton diversity (measured as Shannon index and with equal values of evenness), showing significant and negative effects only in both metacommunities receiving asynchronous nutrient addition (Fig. 4c, e). Since phytoplankton richness did not change once *Cryptomonas* went extinct, a correlation between phytoplankton richness and RUE was inapplicable. RUE also varied with time, showing significant differences only in metacommunities with asynchronous nutrient addition (Online Resource Fig. S5, Fig. 4a). The treatment combination HA resulted in a constant decrease of the ratio (Fig. 4a *r* = − 0.93, *p* < 0.001, Bonferroni-corrected *p* < 0.001), whereas it reached a stabilization point on the fourth week in the LA treatment (Fig. 4a *r* = − 0.9, *p* < 0.001, Bonferroni-corrected *p* < 0.001). In the HS (Fig. 4a *r* = − 0.34, *p* = 0.13, Bonferroni-corrected *p* = 0.26) and LS (Fig. 4a *r* = − 0.29, *p* = 0.19, Bonferroni-corrected *p* = 0.38) treatments, time did not significantly affect the RUE.

## Discussion

The factors tested in this experiment resulted in species dynamics which were mainly propelled by the spatiotemporal heterogeneity of nutrient addition and by time. As expected (h1), not only primary producer diversity but also grazer diversity were positively affected by the asynchronous addition of nutrients; however, this effect disappeared by the end of the experiment. Interestingly and contrary to our expectations (h2), the different levels of connectivity did not affect the species´ dynamics. Nonetheless, greater dissimilarities among patches resulted from the asynchronicity of nutrient addition, in accordance with our hypothesis (h3) and the biomass ratio between zooplankton and phytoplankton decreased over time in the same treatments, showing inverted relationships with zooplankton and phytoplankton diversities (h4).

Independent of local or regional scale, asynchronous nutrient addition promoted a faster increase in phytoplankton Shannon diversity than the synchronous supply after the adaptation phase. However, these effects were transient and time-dependent, with values converging on the fourth week of the experiment. Whether the effects are persistent or transitory are known to be dependent on the competitive abilities of the metacommunity species (Limberger and Wickham 2012b). In our experiment, we explain the transitory effects of asynchronous nutrient supply elucidating the species´ traits and species abundance over time. Since autotroph richness did not change after the *Cryptomonas* sp. loss in the first week of the experiment, the high diversity promoted by asynchronous nutrient addition was mostly related to evenness. The initial lower values of Shannon diversity and evenness on HS and LS treatments were related to the fast growth of *Synechococcus* sp., which was the dominating species in these treatment combinations until the middle of the experiment. Therefore, we could imply that this species was the best competitor under our experiment conditions. As nutrients were often available in all patches due to synchronous nutrient addition, it may have promoted the faster growth of these organisms. Our findings are in line with other experimental studies, as well as the real situation of cyanobacteria blooms, which have attributed rapid responses of *Synechococcus* sp. to high nutrient availability (Phlips et al. 1999; Chung et al. 2011). However, this growth was not persistent, declining after reaching the maximum abundance on the second week. This decline was parallel to the increase in abundance of the other two autotrophs species, *Chlamydomonas* sp. and *Desmodesmus abundans*. In contrast to *Synechococcus* sp., these species were better colonizers, establishing themselves later in the metacommunities. Therefore, diversity changes were time-dependent, initially with lower values due to the dominance of the best competitor (lower evenness), and finally increasing due to the increase of species evenness.

In contrast to diversity effects, the abundance of at least three autotrophs species were more even in the LA and HA treatments from the beginning of the experiment onwards. Our results suggest that the nutrient load to different patches at different times succeeded in creating spatiotemporal nutrient heterogeneity, which prevented the best autotrophic competitor from dominating in all four patches. Addition of high nutrient concentrations in one patch favored *Synechococcus* sp. to succeed locally, but the asynchronicity of nutrient supply intensified the spatial heterogeneity of nutrients, which prevented the best competitor from dominating over time. Moreover, this nutrient loading on a patch rotation basis, promoted higher autotroph diversity not only at the regional scale, but also at the local scale. The well-known hypothesis that higher diversity is promoted by habitat heterogeneity (Simpson 1949; MacArthur and Wilson 1967) has been widely addressed in a metacommunity context (Chesson 2000; Leibold et al. 2004; Davies et al. 2009; Pedruski and Arnott 2011; Hamm and Drossel 2017). In previous work, adding nutrients in a pulsed fashion successfully induced temporal heterogeneity among the interconnected patches, allowing more species to coexist in the metacommunity (Di Carvalho and Wickham 2019). Despite evidence supporting the positive relation between spatiotemporal heterogeneity and species coexistence in phytoplankton communities (de Souza Cardoso et al. 2012), grasslands (Questad and Foster 2008) and in vertebrates (Ivan et al. 2018), few studies have focused on interactive effects of the spatiotemporal aspects of heterogeneity and connectivity.

It has been suggested that species diversity will be highest as a result of niche differentiation at the regional scale, due to the development of habitat heterogeneity (Mouquet and Loreau 2002). In our experiment, the heterogeneity introduced by asynchronous nutrient addition was a much stronger driver of diversity that was connectivity. Higher zooplankton diversity was encountered at the regional than at the local scale, independent of connectivity. While different species could dominate in different patches, even the low connectivity used in our experiment was sufficient to prevent single-species dominance at the regional scale. This lack of single-species dominance was despite the low connectivity treatment allowing migration only 4 h week^−1^. In addition, while asynchronous nutrient addition did not raise average diversity at the local level, there was a strong positive effect at the regional scale over the middle 4 weeks of the experiment.

The lack of connectivity effects on diversity was unexpected. Previous work with similar (though not identical) model communities has found the difference between allowing dispersal for 4 h week^−1^ or 48 h week^−1^ sufficient to produce differences in diversity (Limberger and Wickham 2012b). Furthermore, the presence and absence of connectivity among fragments have already been compared, highlighting the importance of species dispersal in increasing or maintaining diversity (Di Carvalho and Wickham 2019). However, a meta-analysis investigating dispersal and environmental variables effects on species coexistence has pointed out that habitat conditions are more important than species dispersal among sites (Cottenie 2005). Additionally, there are a number of empirical studies that did not find important effects of connectivity on the regional species diversity (Cadotte 2006b, a; Davies et al. 2009). With rapidly-dispersing species, considerable care evidently needs to be taken when defining low- and high connectivity treatments.

The importance of species composition became clearer when the responses of individual species were examined. Despite the rescue effect observed at the regional scale, some species disappeared locally and went extinct after the second week in the synchronous treatments, and 1 week later in metacommunities with asynchronous supply (*Stylonychia* sp. and *Halteria* sp.). The rarity of species observed towards the end of the experiment may explain why the nutrient addition effects were transitory, with synchronous and asynchronous diversity results converging on the fifth week. *Synchaeta oblonga* was nearly extinct from the third week on in all metacommunities, not showing any significant difference among the treatments. The fact that our artificial microcosms were not supplied with a larger species pool made it not possible for a species to return when it went extinct. Aside from *Synchaeta*, all other species of grazers were positively affected by nutrient asynchronicity, either transiently (*Coleps hirtus*, *Cyclidium* sp., *Halteria* sp., *Paramecium bursaria* and *Stylonychia* sp.) or persistently (*Lepadella* sp.). Interestingly, the positive effect of synchronous nutrient addition on *Coleps hirtus*, *Cyclidium* sp. and *Paramecium bursaria*, only appeared on the fourth week, when the abundances of prey species were more even, and the dominance of *Synechococcus* less. It suggests that higher prey diversity does not necessarily result in benefit for the consumers if the non-optimal prey (in our experiment *Synechococcus)* increases in abundance.

Ecologists have long recognized that beta diversity patterns are important to understand species dynamics across sites, and that higher beta diversity is positively related to habitat heterogeneity (MacArthur and Wilson 1967; Kohn and Leviten 1976; Soininen et al. 2007; Heino 2011). In our experiment, we could confirm the assumption that asynchronous nutrient addition would lead to higher beta diversity. Interestingly, the synchronous nutrient addition also promoted dissimilarities among patches, however, at a lower speed, indicating that even under equal abiotic conditions, the patches of a metacommunity might develop differently.

Resource use efficiency decreased in all four treatment combinations over time, with significant effects in LA and HA treatments. Further investigation of the correlation between RUE and species parameters (diversity, richness and evenness) revealed that RUE was negatively related to phytoplankton diversity, as well as evenness (Fig. 4c, e), and positively related to zooplankton richness (Fig. 4d). Therefore, the decrease in RUE over time is explained by the increase in primary producer evenness together with the decrease in grazer diversity. As has long been suggested, higher biodiversity of species is positively related to the resource use efficiency of the ecosystem (Tilman et al. 1997b; Cardinale et al. 2006). However, we observed a RUE decrease, especially in HA and LA metacommunities, under high phytoplankton diversity. This result is better explained by the individual contribution of each species to the absolute biomass in the metacommunities. The increase in the *Chlamydomonas* sp. and the decrease in the *Paramecium bursaria* triggered the decrease of RUE in our system. Low resource use efficiency of zooplankton is related to high phytoplankton richness dominated by cyanobacteria in the field, consistent with the prediction that low food quality of phytoplankton could result in low RUE, even under high phytoplankton diversity (Hassett et al. 1997; Filstrup et al. 2014).

To conclude, we found significant effects of nutrient variability on grazers and primary producers. Diversity and richness were affected by nutrient availability, independently of the spatial scale for phytoplankton and only at the regional scale for zooplankton. We confirmed our prediction that asynchronicity in nutrient supply creates more favorable conditions for species co-occurrence. The spatiotemporal heterogeneity of nutrient availability (LA and HA) treatments allowed different species to survive more efficiently than synchronous nutrient addition, but these differences disappeared towards the end of the experiment. The mechanisms behind the transitory effects of nutrient addition were related to the competitive and colonization abilities of the prey species, and to the artificial nature of our microcosm experiments. Contradicting some studies (Tilman et al. 1997a; Abonyi et al. 2018), we found that the positive relation between resource use efficiency and biodiversity is not always straightforward. In our experiment, the likely explanation for the decrease in resource use efficiency, even under high biodiversity, was the mixture of food quality represented by the phytoplankton species. Our findings highlight the importance of looking beyond diversity or richness, considering the actual species composition as well. In our system, habitat heterogeneity was highly important for the co-occurrence of species, preventing the best competitor from dominating. We recognize the limit of our experiment in predicting natural metacommunities, but we also note that this was not the goal of our work. Even though experiments using artificial microhabitats and microbial species are much simpler than natural ecosystems, they enable ecological theory to be easily tested and monitored with replication of investigations (Lawton 1995; Daehler and Strong 1996; Jessup et al. 2004). We further believe that this research could open new ways of testing fragmentation and eutrophication in a microcosm scale, using different degrees of connectivity and different species composition.

## Electronic supplementary material

Below is the link to the electronic supplementary material.

Supplementary file1 (DOCX 871 kb)
