# Supplementary material for: Does spatiotemporal nutrient variation allow more species to coexist?
Source: Oecologia. 2020 Oct 24;194(4):695–707. doi: 10.1007/s00442-020-04768-9 (PMC7683490; doi:10.1007/s00442-020-04768-9)
Supplement: Supplementary file 1 — Supplementary file1 (DOCX 871 kb) [file 442_2020_4768_MOESM1_ESM.docx]

Electronic Supplemental Material for “Does spatiotemporal nutrient variation allow more species to coexist?”

Josie Antonucci Di Carvalho and Stephen A. Wickham

correspondence: [josie.carvalho@stud.sbg.ac.at](mailto:josie.carvalho@stud.sbg.ac.at)

**Table S1** Statistical analyses and the respective packages and functions used

| Statistical analyses | Package | Function |
| --- | --- | --- |
| Shannon Wiener index (H) | Vegan | diversity |
| Richness (S) | R base | apply |
| Evenness as Pielou´s index (J) | - | J 🡨 H/log(S) |
| Bray Curtis | Vegan | vegdist |
| Test of significance of nutrient addition and connectivity over time using two way ANOVA with repeated measures: | | |
| Shannon Wiener index (H) | Two way ANOVA with repeated measures R Stats | aov |
| Richness (S) | R Stats | aov |
| Evenness (J) | R Stats | aov |
| Bray Curtis | R Stats | aov |
| Tukey´s post-hoc tests: | | |
| Shannon Wiener index (H) | nlme/ lsmeans | lme/ lsmeans |
| Richness (S) | nlme/ lsmeans | lme/ lsmeans |
| Evenness (J) | nlme/ lsmeans | lme/ lsmeans |
| Bray Curtis | nlme/ lsmeans | lme/ lsmeans |


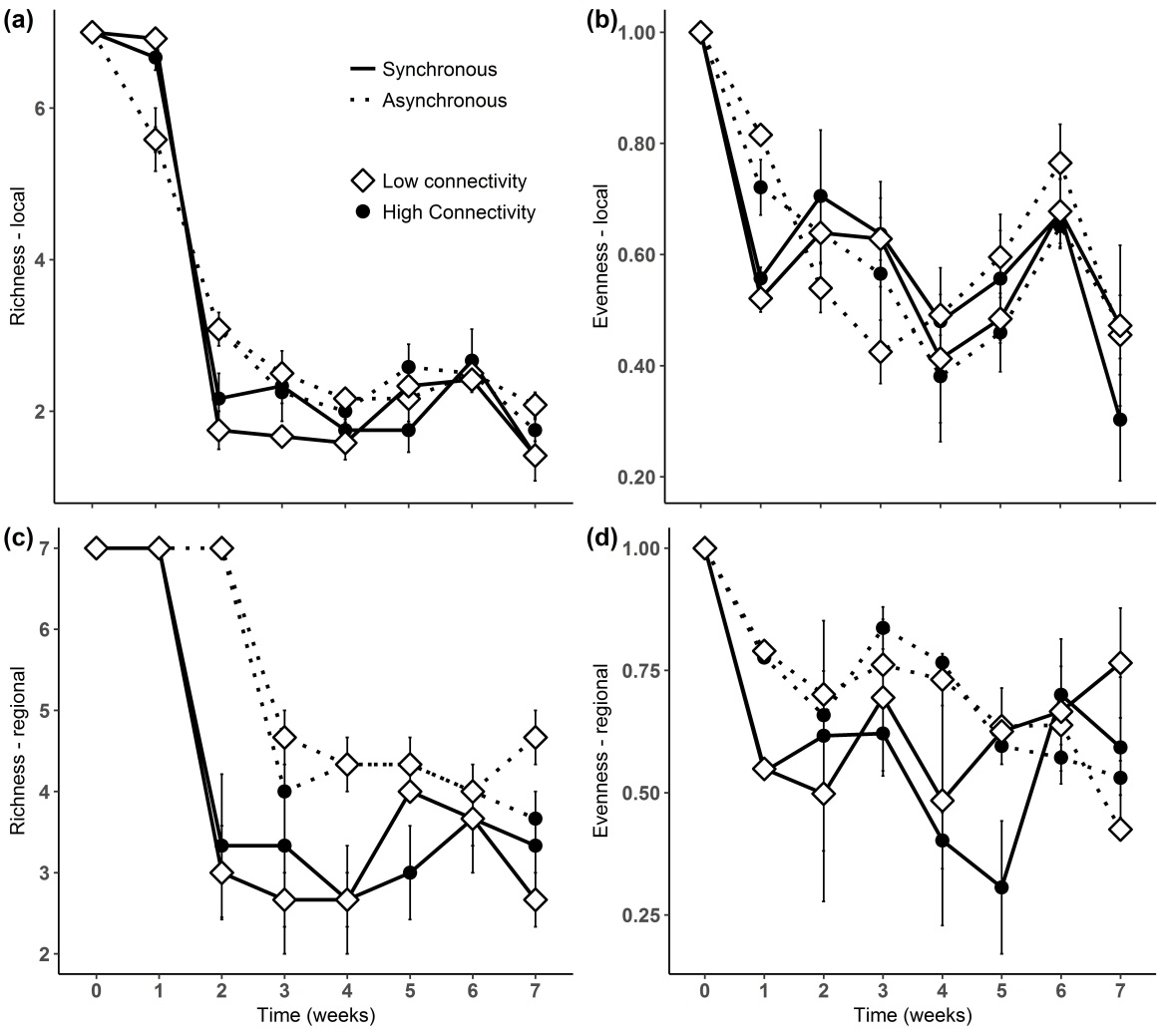


**Fig. S1** Richness and evenness of zooplankton at local and regional scales. (a) Local richness; (b) Local evenness; (c) Regional richness; (d) Regional evenness. In the graphs, low connectivity is represented by opened diamonds; high connectivity is represented by closed circles; synchronous nutrient addition is represented by solid lines and asynchronous nutrient addition is represented by dotted lines. Values are mean ± SE, n = 3


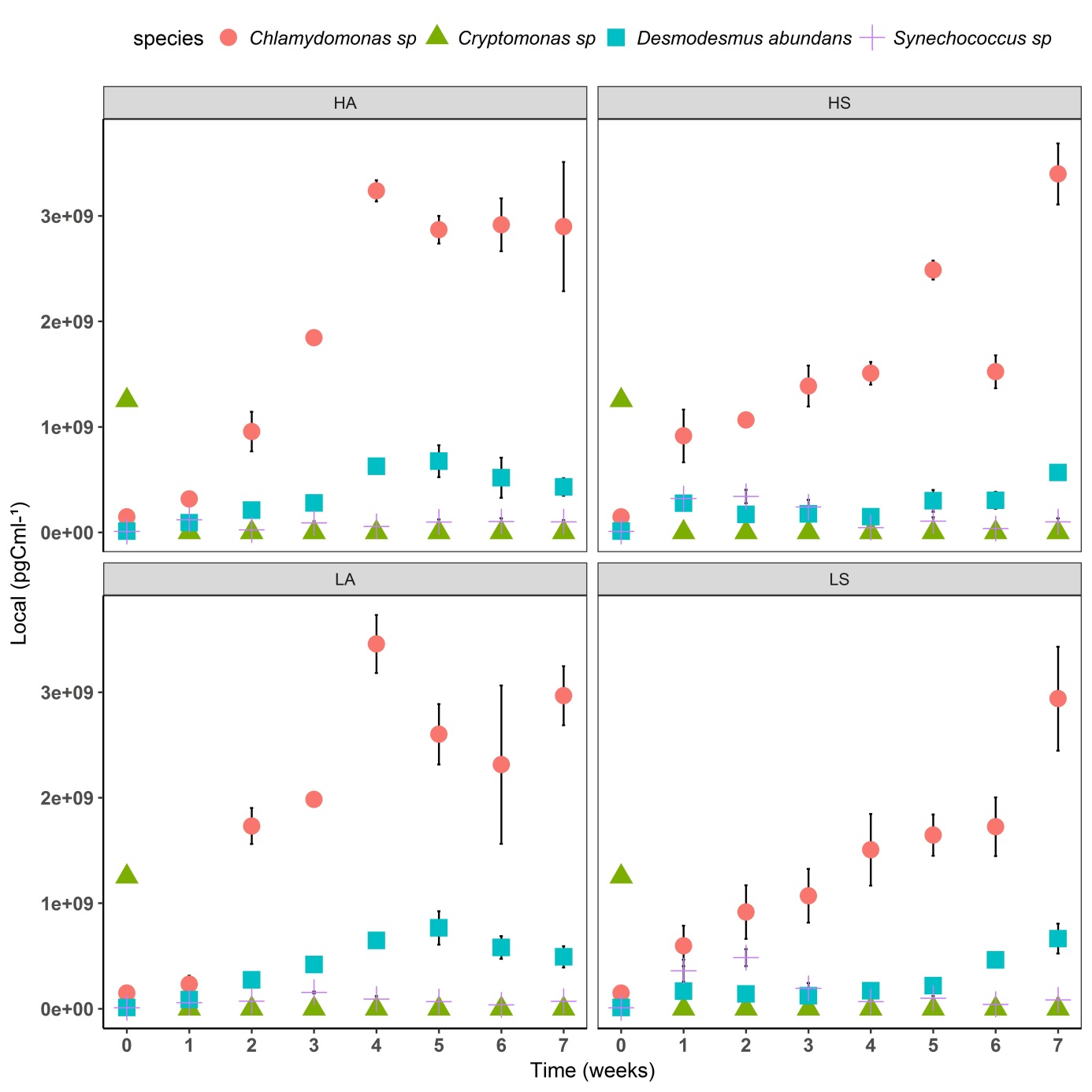


**Fig. S2** Biomass of the four species of phytoplankton. HA = high connectivity with asynchronous nutrient addition; HS = high connectivity with synchronous nutrient addition; LA = low connectivity with asynchronous nutrient addition; LS = low connectivity with synchronous nutrient addition. Values are mean ± SE, n = 3.


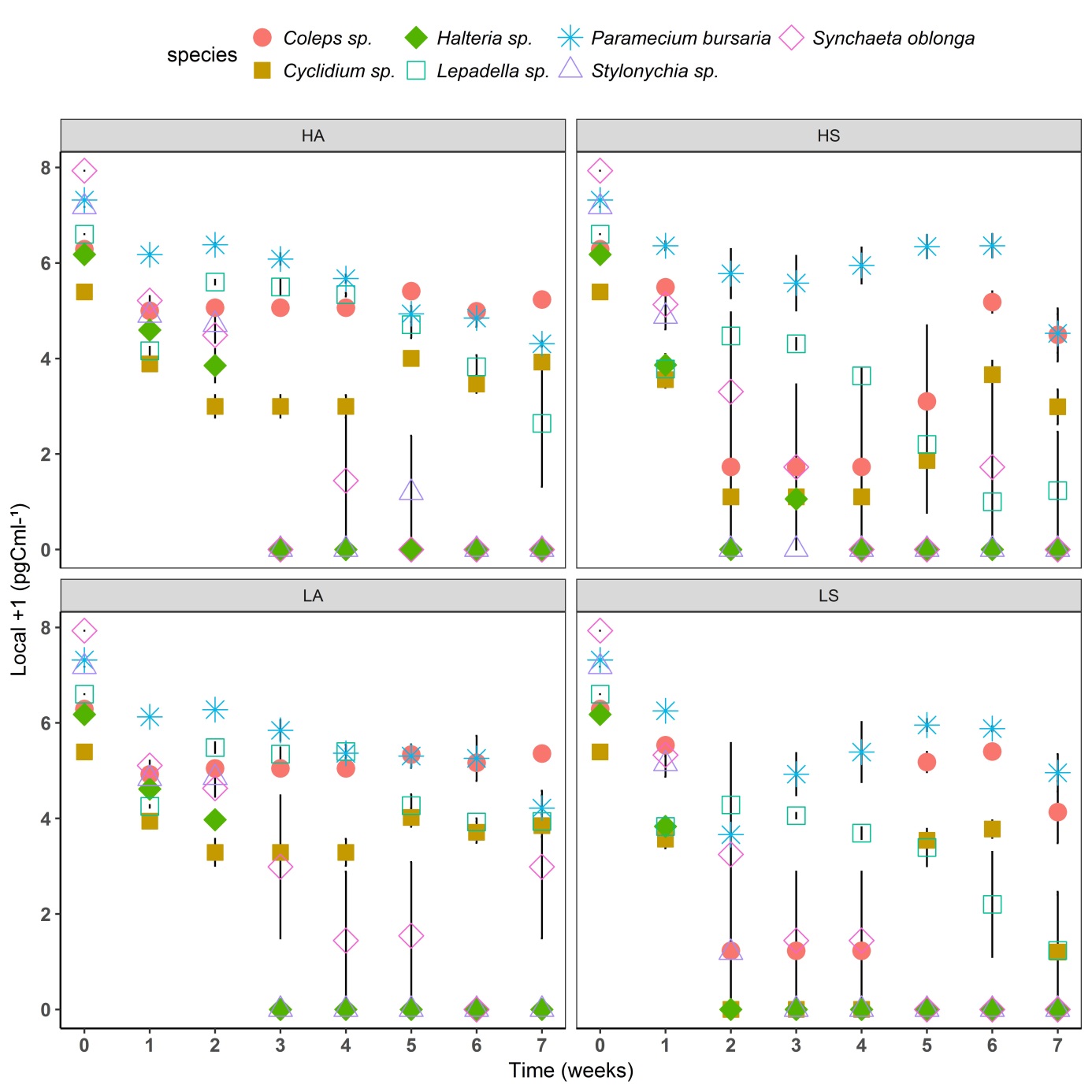


**Fig. S3** Absolute biomass of the seven species of zooplankton. HA = high connectivity with asynchronous nutrient addition; HS = high connectivity with synchronous nutrient addition; LA = low connectivity with asynchronous nutrient addition; LS = low connectivity with synchronous nutrient addition. Values are mean ± SE, n = 3. Note log scale used in panel (log_10_ + 1).


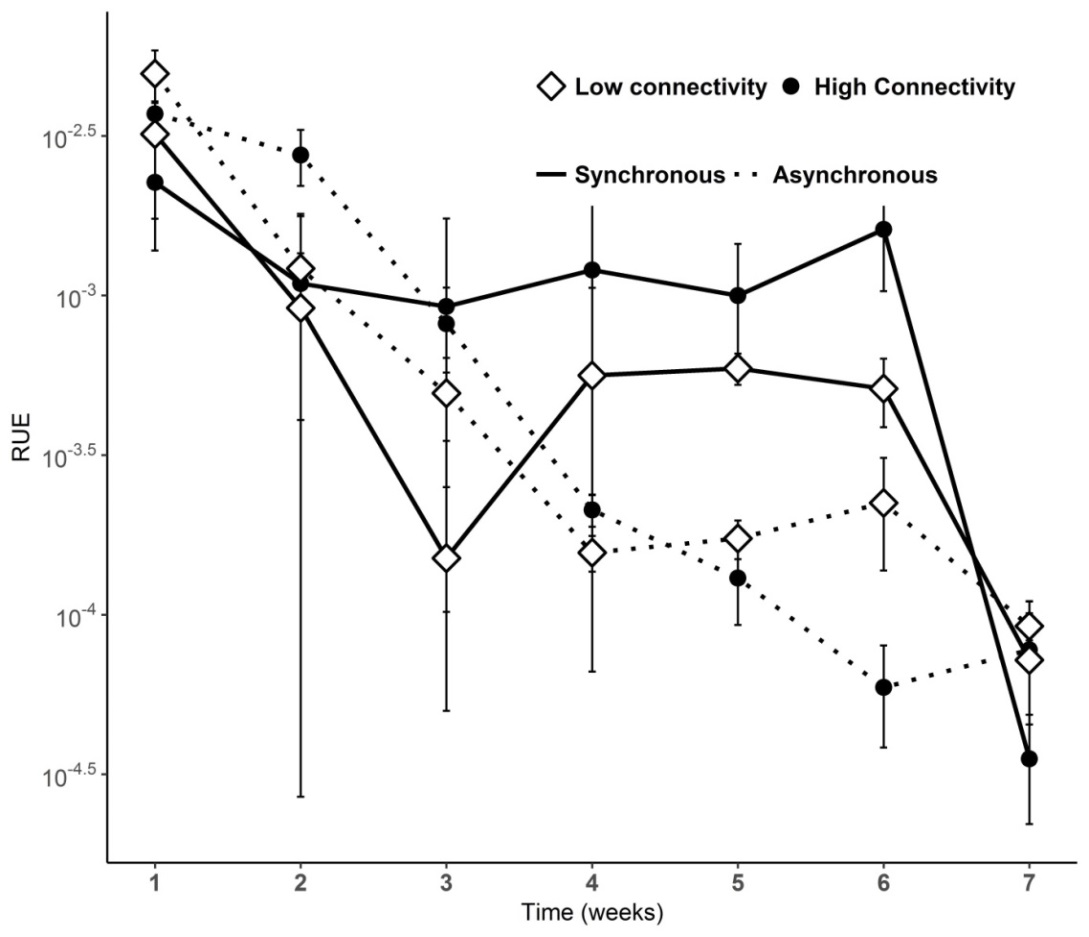


**Fig. S4** Biomass ratio between zooplankton and phytoplankton. In the graphs, low connectivity is represented by opened diamonds; high connectivity is represented by closed circles; synchronous nutrient addition is represented by solid lines and asynchronous nutrient addition is represented by dotted lines. Values are mean ± SE, n = 3


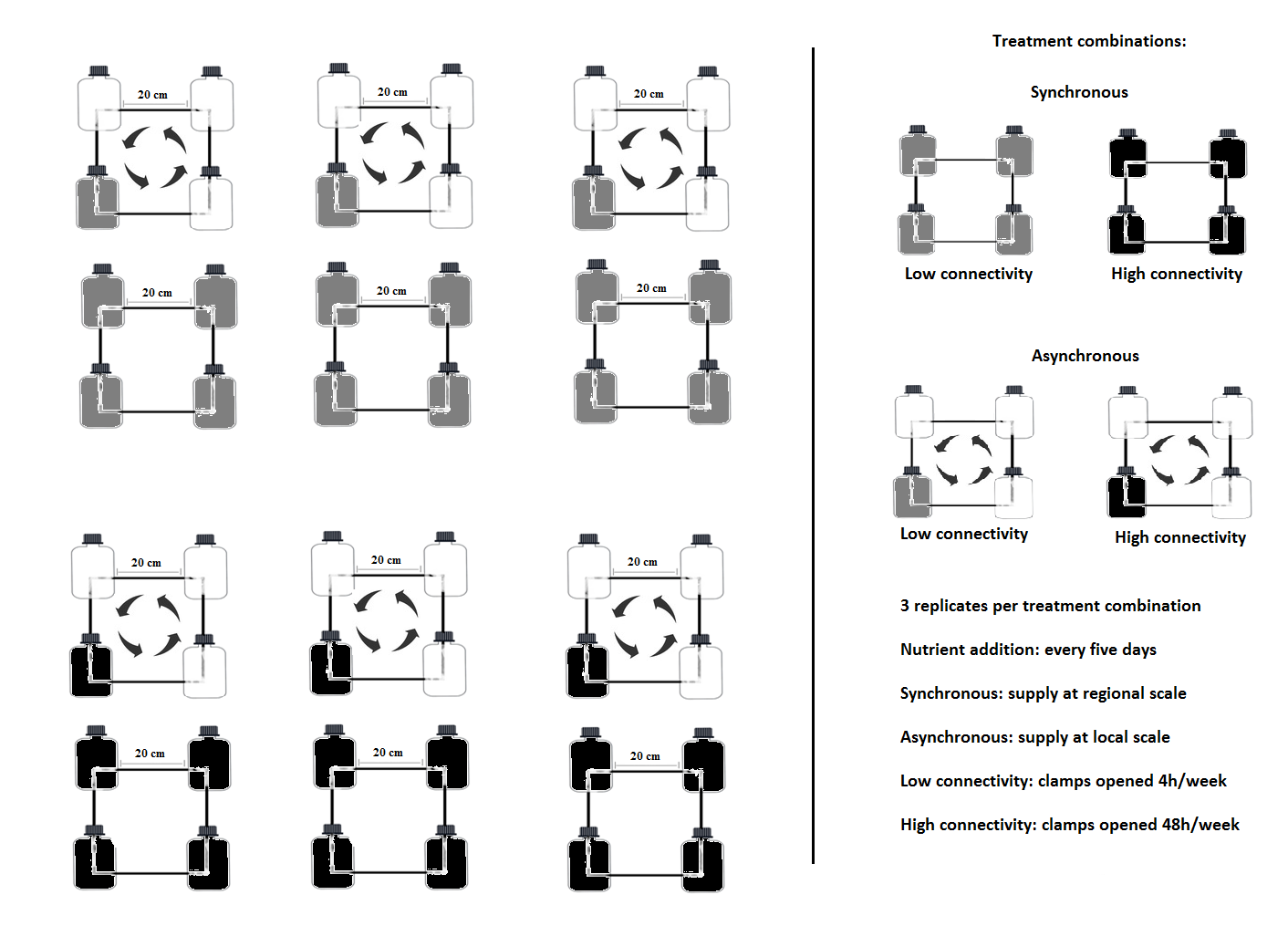


**Fig. S5** Experiment design (drawn picture)
